# Pilot test of Consensus Reporting Items for Studies in Primary care (CRISP)

**DOI:** 10.1017/S1463423623000634

**Published:** 2023-12-19

**Authors:** Elizabeth Sturgiss, William R. Phillips

**Affiliations:** 1 School of Primary and Allied Health Care, Monash University, Melbourne, VIC, Australia; 2 Department of Family Medicine, University of Washington, Seattle, WA, US

**Keywords:** consensus, primary care, reporting guidelines, research, research impact

## Abstract

The Consensus Reporting Items for Studies in Primary care (CRISP) provides a new research reporting guideline to meet the needs of the producers and users of primary care (PC) research. Developed through an iterative program of research, including investigators, practicing clinicians, patients, community representatives, and educators, the CRISP Checklist guides PC researchers across the spectrum of research methods, study designs, and topics. This pilot test included a variety of team members using the CRISP Checklist for writing, revising, and reviewing PC research reports. All or most of the 15 participants reported that the checklist was easy to use, improved research reports, and should be recommended by PC research journals. The checklist is adaptable to different study types; not all items apply to all reports. The CRISP Checklist can help meet the needs of PC research when used in parallel with existing guidelines that focus on specific methods and limited topics.

## Background

The Consensus Reporting Items for Studies in Primary care (CRISP) is a reporting guideline developed by and for primary care (PC) research (Phillips *et al*., 2023). Investigators and users of research across many fields recognize the need to improve research reporting (Glasziou *et al.*, 2014; Moher, 2018). Many have developed guidelines to improve study reports’ quality, validity, and completeness, improving the dissemination, translation, and implementation of new knowledge and reducing research waste (Chalmers and Glasziou, 2009).

The EQUATOR Network catalogs more than 500 guidelines for reporting health research (https://www.equator-network.org). Most address specific research methods and study designs. In addition, many target particular disciplines, limited topics, or focused specialties. However, no published guideline focuses directly on PC’s defining features and perspectives.

PC is a unique clinical specialty and discipline with its own research perspectives and methods (Kidd, 2015). As a result, PC investigators use a variety of study designs and reporting guidelines to cover the breadth of their interests, methods, and topics. The CRISP Checklist complements these method-specific guidelines to enhance the reporting, dissemination, and application of PC research findings and results (Phillips *et al.*, 2023).

PC research also aspires to include the voices and serve the needs of a diverse community, including investigators, practitioners, patients, communities, educators, and policymakers (Phillips *et al.*, 2023). The CRISP guidelines engaged all these groups throughout its development process.

The CRISP guidelines were developed through an iterative program of research (Phillips *et al*., 2023). Our initial survey of the international, interdisciplinary PC research community documented the need for improved reporting of PC research (Phillips *et al*., 2021a). Systematic literature review found no other guideline efforts (Phillips *et al*., 2021b). We then conducted another survey focused on practicing clinicians and their needs (Phillips *et al*., 2021c). From each study, we collated suggested reporting items for PC research. Finally, we conducted a 2-round Delphi study to identify and prioritize the essential items for PC research reports (Sturgiss *et al.*, 2022). We also crafted an Explanation and Examples Report (Phillips *et al*., 2023) to provide further details and context on the appropriate use of the CRISP Checklist across study designs and research topics.

We conducted this pilot test of the draft CRISP Checklist in July – August 2022 to member-check the development process, gather final feedback on the CRISP documents, and test the usability of the CRISP Checklist.

## Methods

We aimed to recruit a diverse survey group of ten PC scientists, clinician-researchers, authors, reviewers, and editors, including multiple disciplines, professions, research methods, and study topics. In addition, we aimed to recruit five comment participants, including patients, study participants, and community partners, to review and comment on the draft CRISP documents but not complete the questionnaire. Using purposive sampling, we invited volunteers via email from the professional networks of the international CRISP Working Group.

We provided each survey participant with the draft documents: CRISP Checklist,^1^ CRISP Statement,^1^ and the CRISP Explanation and Example Report.^11^ Each researcher wrote or revised a PC research report using the CRISP Checklist and returned the completed checklist. Each also completed an anonymous online survey that asked about their demographic characteristics, research training, and experience, the type of research report considered, their experience with using the CRISP process, and their attitudes about using CRISP in PC research reporting. We collected structured data with Yes/No and multiple-choice questions, Likert-like scales, and short free-text comments. The survey was anonymous, open, offered no incentive, and conducted using Qualtrics XM software (Qualtrics, Seattle, WA).

The comment participants provided free text comments via email and did not complete the online questionnaire.

The Monash University Human Research Ethics Committee approved the study. Participants gave informed consent before they proceeded with the survey.

## Results

We invited 12 PC survey participants and received completed questionnaires from 10 (83% response rate). All survey respondents completed all parts of the survey for *n* = 10. Because of the small number, we report results as numbers of respondents.

We also invited five commenters and received comments from all 5 (100% response rate).

Survey respondents came from four nations: USA 3, the Netherlands 3, Australia 2, and Canada 1. Six were women, three described themselves as minorities in their countries, two as Indigenous or First Nations people, and four reported first languages other than English.

By self-reported profession, six were general practice/family medicine physicians, two social workers, one mental health professional, and one other PC physician. Five had research doctoral degrees, and three had master’s degrees. Six described their research experience as advanced, two as intermediate, and two as novice. Eight had authored PC research reports published in peer-reviewed journals. Eight had experience using other reporting guidelines. They filled various roles in PC research (most reported multiple roles): researcher/scientist 6, reviewer 5, trainees 3, clinician-researcher 2, patient 2, community representative 1, and journal editor 1.

They used the CRISP Checklist^1^ while writing or revising different types of research: four quantitative studies, two qualitative studies, two mixed methods, one systematic review, and one other.

Eight survey respondents reported they spent 25–60 extra minutes using the CRISP Checklist to prepare their reports (median 30 min, mean 36.2 min).

We summarize the pilot study participants and questionnaire responses in Table 1. Overall, this diverse group of PC researchers found the CRISP Checklist helpful, well designed, relevant to their studies, and easy to use. Half made changes as a result of using the CRISP Checklist that they felt improved their research reports. All ten respondents said they would recommend the CRISP Checklist to their PC research colleagues and eight plan to use it themselves in the future. All felt it would be appropriate for PC research journals to suggest authors use the CRISP Checklist, but most thought it should be recommended rather than required, at least at this stage in its implementation.

**Table 1.** Results of CRISP checklist pilot study

Pilot study participants	Participants		
	Survey	Free-text comment	Total
Researcher authors	7	0	7
Patients	2	0	2
Community representatives	1	1	2
Reviewers	0	4	4
Total respondents	10	5	15
Survey questions	Yes	No	**Total**
Format			
Are the instructions sufficient?	9	1	10
Is the layout user-friendly?	10	0	10
Is the language in the reporting items clear enough?	10	0	10
Content			
How relevant are the checklist items? (Very Relevant 2, Relevant 8, Not Relevant 0)	10	0	10
Is the length of the checklist (# of items) appropriate?	8	2	10
Checklist contribution			
What was the effect of using the CRISP Checklist on your paper? (CRISP made the paper: Better 5, No change 1, Worse 0, Other 4)			10
CRISP use in the future			
How likely are you to use the CRISP Checklist in the future? (Very Likely 3, Likely 5, Not Likely 2)	8	2	10
Would you recommend checklists to other PC researchers?	10	0	10
Would it be reasonable for a journal to SUGGEST the use of this checklist to authors?	10	0	10
Would it be reasonable for a journal to REQUIRE the use of this checklist of authors?	3	7	10

Participants valued the potential of the CRISP Checklist at multiple stages of the research process: planning studies 5, reviewing reports 5, and teaching research 2.

Several survey and comment participants offered comments:

“Designing studies and writing protocols – if you don’t collect the relevant data, you can’t report it!”

“I could see this as a helpful teaching tool for newer researchers in the field and patient partners. It helps break up the writing of a report or manuscript to make it more manageable.”

“As one gets more experience using the checklist to guide study planning and writing, little extra time will be required, as the learning and process improvement will be “baked in.”

“CRISP users will become better researchers and better writers.”

“Can also be used by authors, reviewers, and editors to … produce a higher quality paper more relevant to primary care.”

The most common concern came from authors noting that some CRISP items did not apply to their specific study.

“Some questions did not apply to my type of study, so could be “optional,” and I thought questions about any quantitative methods used should be added.”

This confusion occurred despite the explanation that not all items applied to all studies or research designs and the instructions for authors to simply check “not applicable” on the CRISP Checklist. For example, qualitative researchers noted items aimed at quantitative research, while quantitative researchers noted items focused on qualitative research.

In addition, four researcher participants used the CRISP Checklist to aid in reviewing PC research manuscripts assigned as usual by peer-reviewed journals. All were mid or senior career researchers. All reported finding the checklist helpful for reviewing a paper. Specifically, CRISP helped these reviewers offer more specific comments and recommendations for the authors and helped to focus the review on the context of PC.

We included two patients and one community representative in the pilot study survey, plus one more representative commented on the draft CRISP documents. They all reported that the CRISP Checklist and reporting items would improve the quality, applicability, and implementation of PC research in practice and patient care. In addition, they felt the CRISP emphasis on describing the research team, the role of patients and community-based clinicians in the research process, and the context of the clinical care would make PC research more relevant to the needs of patients and communities.

## Discussion

The Working Group used these pilot results to guide their final editing of the CRISP Checklist and Explanation and Example Report (Phillips *et al*., 2023).

This was a small survey of a targeted group of volunteers with a risk of bias. However, we value the diverse voices recruited for this pilot test and feel it helps validate our inclusive process for guideline development, designed to meet the needs of the great variety of people and communities involved in PC research. Only broader use across the PC research community can adequately test the usability and value of the CRISP Checklist (Phillips *et al.*, 2023).

Despite these limitations, this pilot study did provide valuable feedback. The quantitative data and comments endorse the content and format, developed through literature review (Phillips *et al*., 2021b), extensive survey research (Phillips *et al.*, 2021b; 2021c), and a formal Delphi process (Sturgiss *et al*., 2022).

Further research should examine the use and effect of the CRISP guidelines among larger numbers of users across the broad landscape of PC research. Important outcome measures would include the rate of use of the checklist by authors, the prevalence of journal suggestions and requirements to use the checklist, the satisfaction of authors and readers, increased quality of research reports (readability, accuracy, completeness, and validity), and improved dissemination and application of research findings/results. The ultimate goals would be better practice, patient care, and health outcomes.

## Conclusion

This small, targeted, pilot study, including a diverse group of PC researchers, demonstrated that the CRISP Checklist is practical, relevant, and valuable for writing and reviewing reports of PC research.

## References

[ref1] Chalmers I and Glasziou P (2009) Avoidable waste in the production and reporting of research evidence. The Lancet 374, 86–89. 10.1016/S0140-6736(09)60329-9 19525005

[ref2] Glasziou P , Altman DG , Bossuyt P , Boutron I , Clarke M , Julious S , Michie S , Moher D , Wager E . (2014) Reducing waste from incomplete or unusable reports of biomedical research. The Lancet 383, 267–276. 10.1016/S0140-6736(13)62228-X 24411647

[ref3] Kidd M (2015) The importance of being different: inaugural Dr Ian McWhinney lecture. Canadian Family Physician 61, 1033–1038. 26668275 PMC4677932

[ref4] Moher D (2018) Reporting guidelines: doing better for readers. BMC Medicine 16, 233. 30545364 10.1186/s12916-018-1226-0 PMC6293542

[ref5] Phillips WR , Gebauer S , Kueper JK , Martinez-Guijosa A , Felzien M , olde Hartman TC , Westfall JM , DeVoe JE , Stewart M , Herbert CP , Green LA , Belle Brown J . (2023) Primary care research: looking back and moving forward with reflections on NAPCRG’s first 50 years. Annals of Family Medicine 21, 456–462. 37748895 10.1370/afm.3009 PMC10519759

[ref6] Phillips WR , Louden DN and Sturgiss E (2021b) Mapping the literature on primary care research reporting: a scoping review. Family Practice 38, 495–508. 33599778 10.1093/fampra/cmaa143

[ref7] Phillips WR , Sturgiss E , Glasziou P , olde Hartman TC , Orkin AM , Prathivadi P , Reeve J , Russell GM and van Weel C . (2023) Improving the reporting of primary care research: consensus reporting items for studies in primary care—the CRISP statement. Annals of Family Medicine 2023 3029. 10.1370/afm.3029. PMC10681700 37788942

[ref8] Phillips WR , Sturgiss E , Hunik L , Glasziou P, olde Hartman T, Orkin A, Reeve J, Russell G and van Weel C. (2021a) Improving the reporting of primary care research: an international survey of researchers. Journal of the American Board of Family Medicine 34, 12–21. 33452078 10.3122/jabfm.2021.01.200266

[ref9] Phillips WR , Sturgiss E , Yang A , Glasziou P , Olde Hartman T , Orkin A , Russell GM and van Weel C . (2021c) Clinician use of primary care research reports. Journal of the American Board of Family Medicine 34, 648–660. 34088824 10.3122/jabfm.2021.03.200436

[ref10] Sturgiss EA , Prathivadi P , Phillips WR , Moriarty F , Lucassen P , van der Wouden JC , Glasziou P , olde Hartman TC , Orkin A , Reeve J , Russell G and van Weel C . (2022) Key items for reports of primary care research: an international Delphi study. BMJ Open 12, e066564. 10.1136/bmjopen-2022-066564 PMC9764621 36535712

